# The integral role of the Black Soldier fly, *Hermetia illucens* L., microbiota in its life history

**DOI:** 10.3389/frmbi.2025.1517715

**Published:** 2025-06-26

**Authors:** Dana Ment, Sapna Mishra

**Affiliations:** Department of Plant Pathology and Weed Research, Agricultural Research Organization, Volcani Center, Rishon LeZion, Israel

**Keywords:** Black Soldier fly, bacterial community, protection, nutrient provision, gut microbiota

## Abstract

The Black Soldier fly (BSF), *Hermetia illucens*, exhibits versatile bioconversion abilities and effectively transforms various waste materials into a nutritious biomass suitable for consumption. The degradation ability of BSF larvae has been attributed to their gut microbiota. Therefore, this review explores the role of the BSF microbiota throughout the BSF life stages in the bioconversion, focusing on the BSF larvae and its microbiota. We reflect on the microbiota’s contribution to life cycle aspects, growth, reproduction, immune response, and waste breakdown. The key points discussed include the gut microbiota in organic waste bioconversion by BSF larvae, the role of microbiota in BSF oviposition and growth throughout its life history, and microbiota’s role in immunity with a specific focus on antimicrobial peptides. Where knowledge gaps were identified for BSF, we provide examples of closely related dipteran insects or insects with well-studied microbiota functioning. The significant role of the BSF gut microbiota is enabling its versatile waste degradation while conferring protection against pathogens and xenobiotic compounds. As such, we discuss the future perspectives that microbiome engineering may offer for BSF.

## 1 Introduction

The larvae of the Black Soldier fly (BSF), *Hermetia illucens* L. (Diptera: Stratiomyidae), exhibit versatile bioconversion abilities and effectively transform various waste materials into a nutritious biomass suitable for consumption (Chia et al., 2018; Spranghers et al., 2017). The degradation ability of BSF larvae has been attributed to their gut microbiota. The microbial communities residing in the gut of BSF larvae possess unique metabolic capabilities, which play a crucial role in the degradation and transformation of organic substrates. Generally, in insects and in BSF, enzymes produced by gut bacteria, such as proteases, cellulases, and lipases, assist in the breakdown of complex organic matter present in the substrate to facilitate the digestion and metabolism of nutrients (Engel and Moran, 2013; Eke et al., 2023). Enhanced nutrient absorption ensures that BSF larvae obtain sufficient energy and nutrients for growth and development, producing abundant biomass.

As BSF larvae feed on organic waste materials it releases antimicrobial peptides and enzymes into the environment (Vogel et al., 2018; Zhang et al., 2021). These compounds help in inhibiting the growth of pathogenic bacteria and other microorganisms present in the substrate. Anti -microbial peptides (AMPs) isolated from BSF larvae have been shown to exhibit potent activity against a wide range of bacteria, including both Gram-negative (e.g., *Escherichia coli*) and Gram-positive species (e.g., methicillin-resistant *Staphylococcus aureus*) (Park et al., 2015). By suppressing the growth of these pathogenic bacteria, BSF larvae may create a more hygienic environment within the substrate that not only helps in preventing the spread of diseases but also facilitates the decomposition of organic matter. As a result, the bioconversion process is accelerated, leading to the production of cleaner, nutrient-rich compost or biomass.

In addition, the presence of a diverse and abundant gut microbiota in BSF larvae acts as a barrier against colonization by pathogenic microorganisms through competition for resources, modulation of the host immune response, and modification of environmental conditions (Eke et al., 2023; De Smet et al., 2018). This helps to maintain the health and integrity of the larval gut ecosystem and contributes to the overall fitness and survival of the BSF larvae. The diverse gut microbiota of BSF larvae also enhances to the biotransformation of various compounds present in the substrate, including the degradation of complex organic pollutants, such as xenobiotics, pesticides, and pharmaceuticals, into less harmful or more easily degradable forms (Lalander et al., 2016; Van der Fels-Klerx et al., 2020).

Xenobiotics present in organic waste materials are persistent and able to contaminate soil, water, and food sources, thus posing risks to ecosystems, and human and animal health. By metabolizing xenobiotic present in organic waste materials, BSF larvae activity reduce the risk of their exposure to humans and animals, as well as contribute to the efficient conversion of waste into valuable biomass (Gold et al., 2018). This enhances the overall waste management process, making it more sustainable and environmentally friendly. In summary, the gut microbiota of the BSF play a crucial role in the efficient conversion of organic waste materials into valuable biomass, highlighting the importance of these microbial communities in the BSF gut into sustainable waste management practices.

The present review specifically explores the role of the BSF microbiota throughout its life stages, highlighting their contribution to various aspects such as growth, sustenance, reproduction, immune response, and waste breakdown. Where knowledge gaps were identified, we provide examples of closely related insects and offer perspectives for future research.

## 2 Functional role of bacterial communities associated with egg to adult

Bacterial communities associated with eggs of BSF play a pivotal role in various aspects of the insect’s life cycle and ecosystem functioning. The bacterial community as a whole may serve multiple functions crucial for egg viability and offspring development and gravid female attraction to oviposition site (De Smet et al., 2018; Zheng et al., 2013). For example, *Bacillus* sp. and *Gordonia* sp. significantly increased the oviposition response of BSF females (Zheng et al., 2013). Similar evidences were found in houseflies, in which provided evidence that adult houseflies use cues from bacterial communities to locate suitable environments for laying their eggs. Thus, through detection and quantification of these bacterial signals, the houseflies ascertain whether a specific substrate is appropriate for egg depositing (Lam et al., 2007). This conserved behavior among dipterans highlights the importance of microbial communities in mediating the ecological interactions between BSF and their environment, particularly in the context of reproductive behavior and oviposition site selection.

There are contradictory evidences of the role of the egg-associated bacteria as a protective barrier against pathogenic microorganisms. Some studies suggest protective role of egg-associated bacterial communities which safeguard eggs from infections, ensuring the survival of the developing larvae. Additionally, BSF eggs-bacterial communities (e.g., Enterococcus, Providencia, Morganella, Buttiauxella) contribute to the decomposition and nutrient cycling processes taking place by the larvae (De Smet et al., 2018; Tegtmeier et al., 2021). As the eggs hatch and larvae emerge, they ingest bacteria along with organic substrates, initiating the breakdown of complex organic matter and facilitating nutrient release (Yu et al., 2011). Furthermore, certain bacteria within these communities may serve as essential symbionts for larvae, aiding in digestion, nutrient absorption, and overall larval development. On the contrary, it has been suggested that bacteria of the species *Bacillus*, *Lysinibacillus* and *Oceanobacillus* did not increase larval performance even at high concentrations (Schreven et al., 2021).

Many insects that foster endosymbionts employ vertical transmission via the egg to assure the transfer of microorganisms (Nyholm, 2020). Vertical transmission within the egg facilitates the proper transfer of symbionts crucial for nutritional or other supportive functions within their hosts. This highlights the overall significance of the bacterial communities associated with eggs to insect fitness, ecosystem nutrient cycling, and environmental sustainability. The following sub-section delves into the overall role of bacterial communities associated with BSF eggs in colony establishment and insect well-being.

### 2.1 Role in oviposition

**Figure 1:**
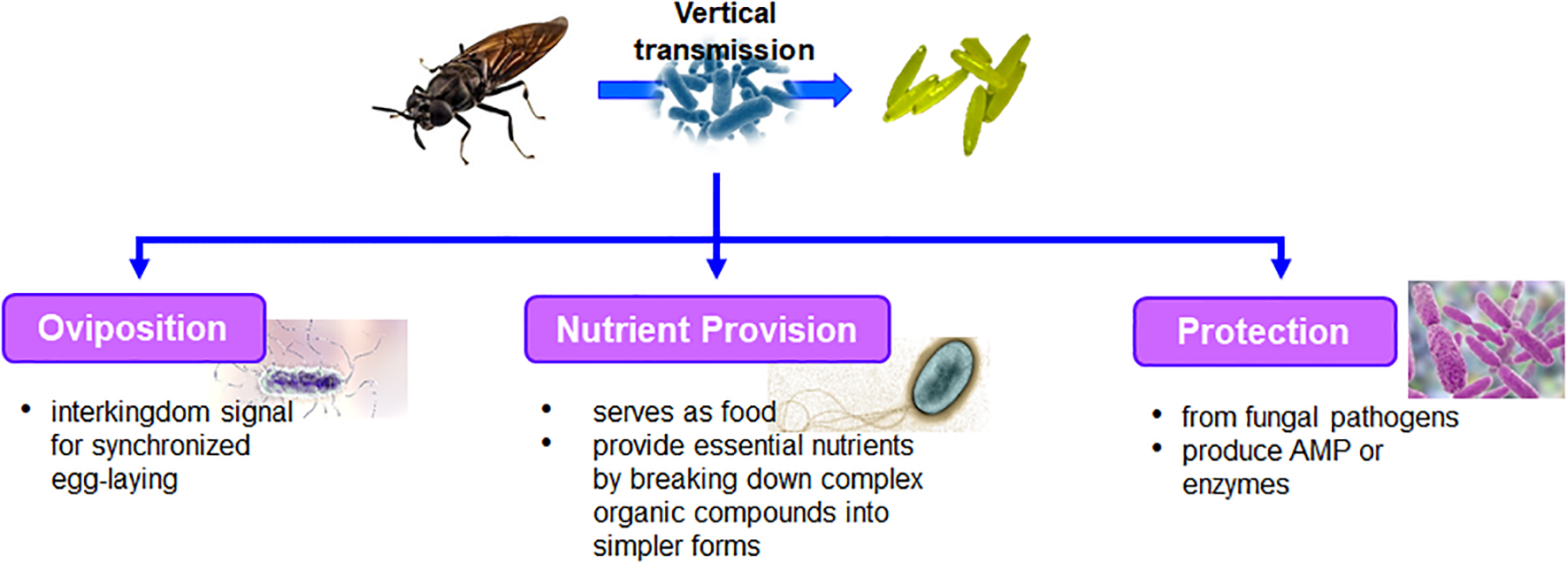
Functional role of bacteria associated with adult, egg and its interaction.

Bacterial communities play a crucial role in the oviposition behavior of insects including BSF, influencing various aspects of their reproductive ecology. Gravid female BSF are known to preferentially deposit their eggs in substrates rich in organic matter (De Smet et al., 2018). These oviposition sites often harbor diverse bacterial communities, which impact insect fitness and reproductive success in several ways. Bacterial symbionts associated with insects may directly influence female oviposition behavior by modulating hormone levels or physiological processes involved in reproductive signaling (**Figure 1**). Zheng et al. (2013) hypothesized that both symbionts are found in the place where insects lay their eggs and the eggs themselves might control how the insects are attracted to that area, colonize in, and eventually become the dominant insect species there. This study also ascertained that using a combination of bacteria, rather than just one species, has varying effects on the behavioral responses of flies, including BSF. Presence of multiple bacterial species in the oviposition sites might enhance the attractiveness of oviposition site to insects.

As literature regarding BSF specifically is scarce, we give some examples from other dipterans. Insects frequently utilize specialized signals to facilitate the development of their progeny, thereby decreasing intra-species competition. Ovipositing tephritid fruit flies release pheromones that deter further egg-laying (Scolari et al., 2021), while female house flies prefer to lay their eggs in close proximity to recently laid eggs of their own kind (Lam, 2010; Lam et al., 2007). Lam et al. (2007) proposed a dynamic ever-evolving signaling mechanism in houseflies that transition from promoting egg laying to its inhibition after a certain period following egg deposition. The inhibition prevents egg deposition by late-arriving females in order to prevent the older progeny from cannibalizing the younger ones, thereby creating a favorable environment for the growth of progeny. This evolving signal was attributed to egg-associated bacteria, *Klebsiella oxytoca* that originated from the female houseflies. *Klebsiella oxytoca* proliferates over time on the surface of deposited eggs, and when it achieves a threshold density, an ovipositional signal to prevent any further oviposition by gravid female houseflies is ensued. This arrangement also allows for synchronous aggregation and offspring deposition that results in optimal colonization densities of even-aged larvae. Though *Klebsiella* was identified in BSF (Gorrens et al., 2021), its effect on egg deposition by females was not studied. The presence of fresh larval frass from BSF suppresses tendency of houseflies to lay eggs (Adjavon et al., 2021). This effect seems to happen because the fresh frass reduces the release of volatile organic compound, apparently used by houseflies use as a cue for finding places to lay their eggs. The change of substrate composition that reduced attractiveness to houseflies for oviposition was attributed to the L strain bacteria of genus *Paenalcaligenes*. It would be interesting if future studies find any relation between volatile of fresh larval frass with the presence and density of *K. oxytoca* in this substrate. Another possible explanation for oviposition deterrence was stated to be the reduction in the number of *Escherichia coli* in the BSF larvae digested substrate, thereby cutting off a potential nutritional source for the housefly larvae (De Smet et al., 2018; Liu et al., 2008).

Two bacterial species, *Ignatzschineria* sp. and *Acinetobacter* sp., isolated from competitors of BSF, the secondary screwworm (*Cochliomyia macellaria*) and the lesser mealworm (*Alphitobius diaperinus*), respectively, caused BSF to avoid laying eggs, leading to fewer eggs being deposited (Zheng et al., 2013). However, two other bacterial species, *Gordonia* sp. (isolated from BSF eggs) and *Providencia* sp. (isolated from the hairy maggot blowfly *(Chrysomya rufifacies*), actually increased BSF egg-laying. These bacteria likely release certain chemicals or unidentified odors that either attract or repel female BSF, guiding them to or away from suitable egg-laying sites. This inter-species communication through signaling molecules is thought to play a significant role in the egg-laying behavior of several insect species.

### 2.2 Role in nutrient provision

The presence of specific bacteria in the substrate can influence the nutritional quality and microbial composition, providing essential nutrients or metabolites that promote egg viability and larval development. Bacteria play a vital role in providing nutrients to insects during egg laying, contributing to the reproductive success and offspring development of various insect species (**Figure 1**). Gravid female BSF often select oviposition sites rich in organic matter that are colonized by diverse bacterial communities (Zheng et al., 2013). The bacterial communities actively break down complex organic compounds into simpler forms and is readily assimilated by the insects. Bacteria within these communities produce enzymes that degrade complex polymers such as cellulose, lignin, and proteins into smaller molecules like sugars, amino acids, and fatty acids, which serve as essential nutrients for egg development and larval growth. Additionally, certain bacteria such as *Lysinibacillus fusiformis* directly serve as food for the newly emerged larvae. Bacteria are essential dietary constituents for developing housefly larvae and aid in their development to adult flies (Lam et al., 2009a). Female houseflies typically lay their eggs on substrates that contain specific bacteria (*Klebsiella oxytoca*). Alternatively, these bacteria can be deposited onto the substrate by the female flies during oviposition. When the eggs hatch, the emerging larvae have access to an ample supply of bacterial food, regardless of the initial quantity of bacteria present at the oviposition site ensuring that the larvae have sufficient nutrition for their development and growth. The maternally-transmitted microbiota of *Drosophila melanogaster* produce the fermentation product acetoin. For instance, the presence of acetoin affects the odor produced by utilizing the substrate or food source, which, in turn, impacts the larvae’s preference for that particular food (Farine et al., 2017). This suggests that the microbiota inherited from the female fly can significantly influence the behavior and feeding preferences of the larvae, highlighting the intricate relationship between the microbiota and the developmental processes of *Drosophila melanogaster*.

### 2.3 Role in protecting against pathogens in oviposition sites

Bacterial communities play a crucial role in protecting insects from fungal pathogens during egg laying, contributing to the overall health and survival of insect populations. Certain bacteria associated with oviposition sites may produce antimicrobial compounds or enzymes that inhibit the growth and proliferation of fungal pathogens, thereby safeguarding the eggs from infection (**Figure 1**). These protective bacteria may colonize the surface of eggs, forming a defensive barrier against fungal colonization, or produce metabolites that suppress fungal growth in the surrounding environment. Bacterial symbionts inhabiting the insect may confer resistance to fungal infections through direct antagonistic interactions or by modulating the insect immune system. The protective role of bacteria is more prominent in insects, including BSF, that lay their eggs in microbe rich environments, where they face constant risk of infection by the residing pathogens (Nyholm, 2020). For instance, Lagria beetles exhibit a symbiotic relationship with several strains of *Burkholderia* bacteria (Flórez et al., 2018). During the process of oviposition, these beetles deposit the bacteria onto the surfaces of their eggs. Once on the egg surface, *Burkholderia* bacteria engage in a unique behavior where they produce a type of antifungal compound called polyketide lagriamide. This lagriamide serves as a protective shield, guarding the beetle larvae against potential fungal infections that could otherwise harm or impede their development. Similarly, the solitary digger wasp *Philanthus triangulum* forms a mutually beneficial relationship with a bacterial symbiont *‘Candidatus* Streptomyces philanthi’ (Koehler et al., 2013). This bacterium plays a vital role in protecting the developing wasp larvae by producing a mixture of at least nine antibacterial compounds on the surface of the cocoon. These compounds collectively act as antibiotics, inhibiting the growth and development of numerous fungi and bacteria. Defensive symbionts, which provide protection to their host organisms, are typically facultative and tend to adapt and coevolve with their local antagonist communities (Engl et al., 2018). This adaptive behavior enables bacterial symbionts to effectively defend their host organisms against evolving threats posed by antagonistic organisms in their surroundings. Lam et al. (2009b) highlights the significant role of bacteria consortia associated with housefly eggs in preventing the growth of fungi on the substrate, which could otherwise compete with housefly eggs for nutrients. Various strategies are employed to suppress fungal growth, such as nutrient competition or antibiosis. The author demonstrated that while no individual bacteria could significantly inhibit the growth of all fungal strains, their collective action as a group proved to be effective in suppressing fungal growth. Among the bacteria studied, *K. oxytoca* exhibited the most significant antifungal activity, indicating its potential not only as oviposition cue for pregnant female houseflies (Lam et al., 2007) but also as a candidate for developing antifungal treatments (Lam et al., 2009b). For instance, the eggs of mealworm beetle *Tenebrio molitor* possess innate defense mechanisms to protect against Gram-positive pathogenic bacteria by producing antibacterial peptide belonging to the defensin family, tenecin 1 (Dubuffet et al., 2015).

## 3 Bacterial communities associated with larvae

One notable characteristic that sets insects apart from mammals and other vertebrates is their process of metamorphosis, which significantly influences the composition and stability of its microbiota. Holometabolous insects, such as flies, harbor distinct gut microbial communities during their larval and adult stages (reviewed by Hammer and Moran, 2019). This variation arises because the gut environment undergoes a complete transformation during pupation, which imposes challenges for the persistence of microbial communities established during the larval stage. Thus, the transition from larvae to adults represents a critical period where gut microbial communities must adapt to the changing conditions to establish and maintain a stable microbiota throughout the insect’s life cycle. Bacteria residing within BSF larvae inhabit various tissues, including the compartments of the digestive tract, hemolymph, fat body, and reproductive organs, where they may serve diverse functions such as nutrient provisioning, immune modulation, and defense against pathogens. Of this, digestive tract of BSF larvae is of utmost importance and harbors a diverse microbial community. The larval gut has intricate structure allowing various functional adaptations, and reflect their remarkable efficiency in nutrient acquisition, and waste processing. The bacterial communities within insect gut also vary considerably in total size, composition, locations inside the gut, and affiliated functions (Callegari et al., 2021; Engel and Moran, 2013). Insects that feed on organic matter or wood, such as BSF and termites, tend to host a greater abundance and diversity of gut microbiota (Eke et al., 2023; Mikaelyan et al., 2017). This suggests that the dietary habits of insects play a significant role in shaping the composition and diversity of their gut microbial communities. Insects consuming organic matter or wood likely benefit from a more diverse microbiota, which may aid in the digestion and utilization of their specialized diets.

In general, the gut microbiota of insects consists of both symbiotic organisms and bacteria acquired from their environment through exposure and feeding. Symbionts can be divided into two categories: obligate and facultative. Multiple endosymbionts can simultaneously inhabit a single host, and provide distinct benefits to insects, such as, improving nutrition, protection against parasites and pathogens, and boosting tolerance to abiotic challenges. Moreover, different symbionts within the same host may collaborate to provide complementary benefits. The level of specialization and adaptation of a symbiont to a host’s intestinal environment is determined by their transmission fidelity. In contrast, the survival of environmentally acquired bacteria in the insect gut is primarily influenced by the physicochemical characteristics of gut compartments, including pH, redox potential, and substrate availability (Callegari et al., 2021; Eke et al., 2023; Mikaelyan et al., 2017). Insects’ gut conditions tend to support specific bacterial species, therefore even though each generation of insects acquires bacteria from the environment independently, they still favor a particular group of bacteria (Eke et al., 2023). This explains why, despite the influence of diet or location on gut microbial communities, a few bacterial taxa are frequently detected in the BSF gut. The recurrence of specific microbial species in the gut communities may stem from direct transmission among insects, selective uptake by the hosts, specific adaptation of microorganisms to colonize the insect guts, or a combination of these factors.

### 3.1 Effect of gut microenvironment

The extent of microbe selection by the host insect is influenced by various physicochemical conditions within their gut, including the transit rate of food, nutrient availability, local pH levels, redox potential, and the presence of immune factors. These factors can vary along the length of the gut, leading to distinct colonization patterns in different gut regions (Powell et al., 2014; Schmidt et al., 2023). In many animals, the microbial density tends to increase from the anterior to the posterior part of the digestive system. This gradient allows the microbiota to avoid direct competition with the host for nutrients, as the anterior gut segments are typically involved in nutrient absorption (Engel et al., 2013). This spatial differentiation in microbial colonization patterns also reflects an adaptation to optimize nutrient utilization and minimize competition between the host and its associated microbial communities.

**Figure 2:**
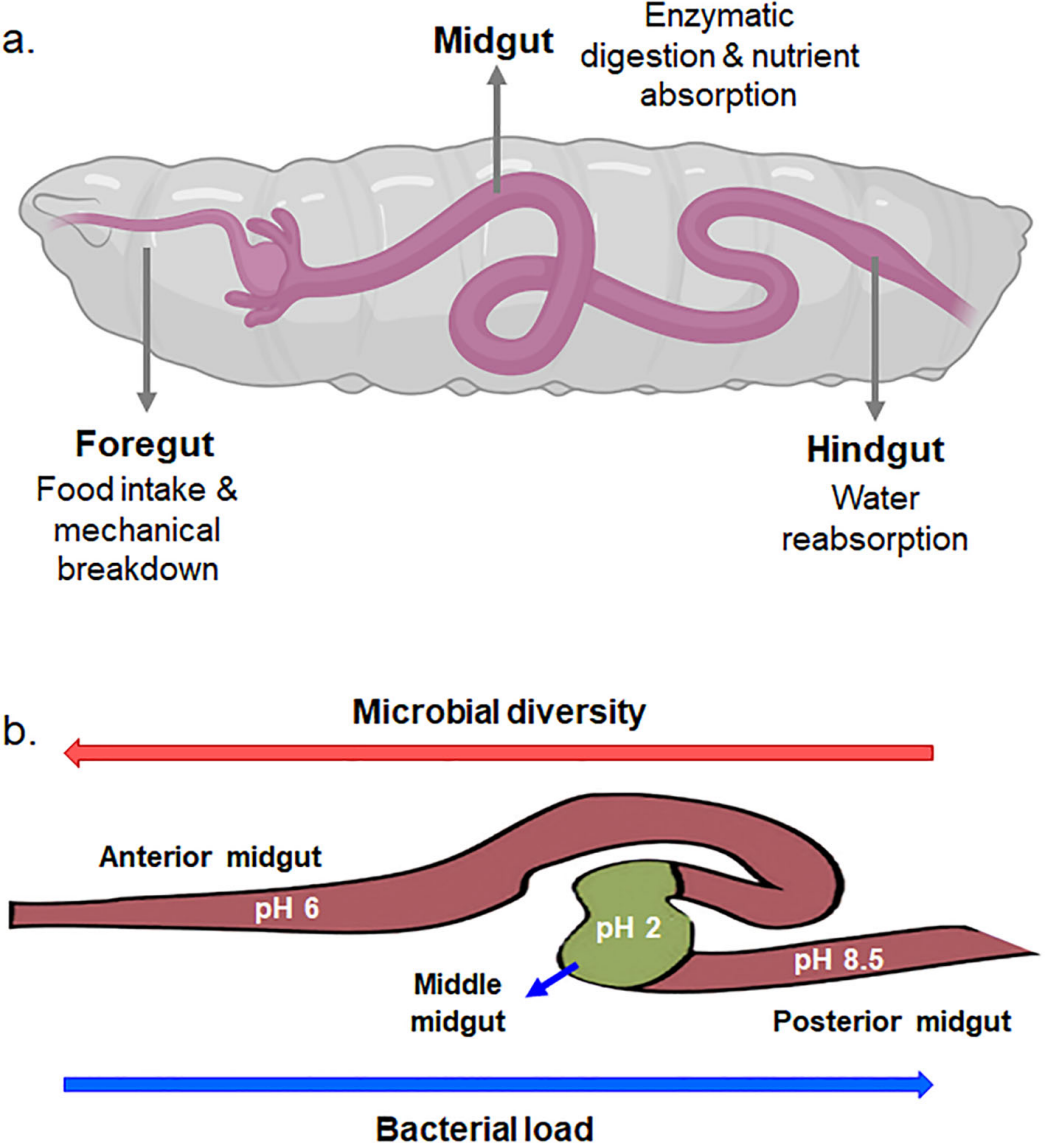
**(a)** Digestive system of BSF larva. **(b)** Role of pH variation in the midgut of BSF larvae in determining microbial load and diversity.

**Figure 3:**
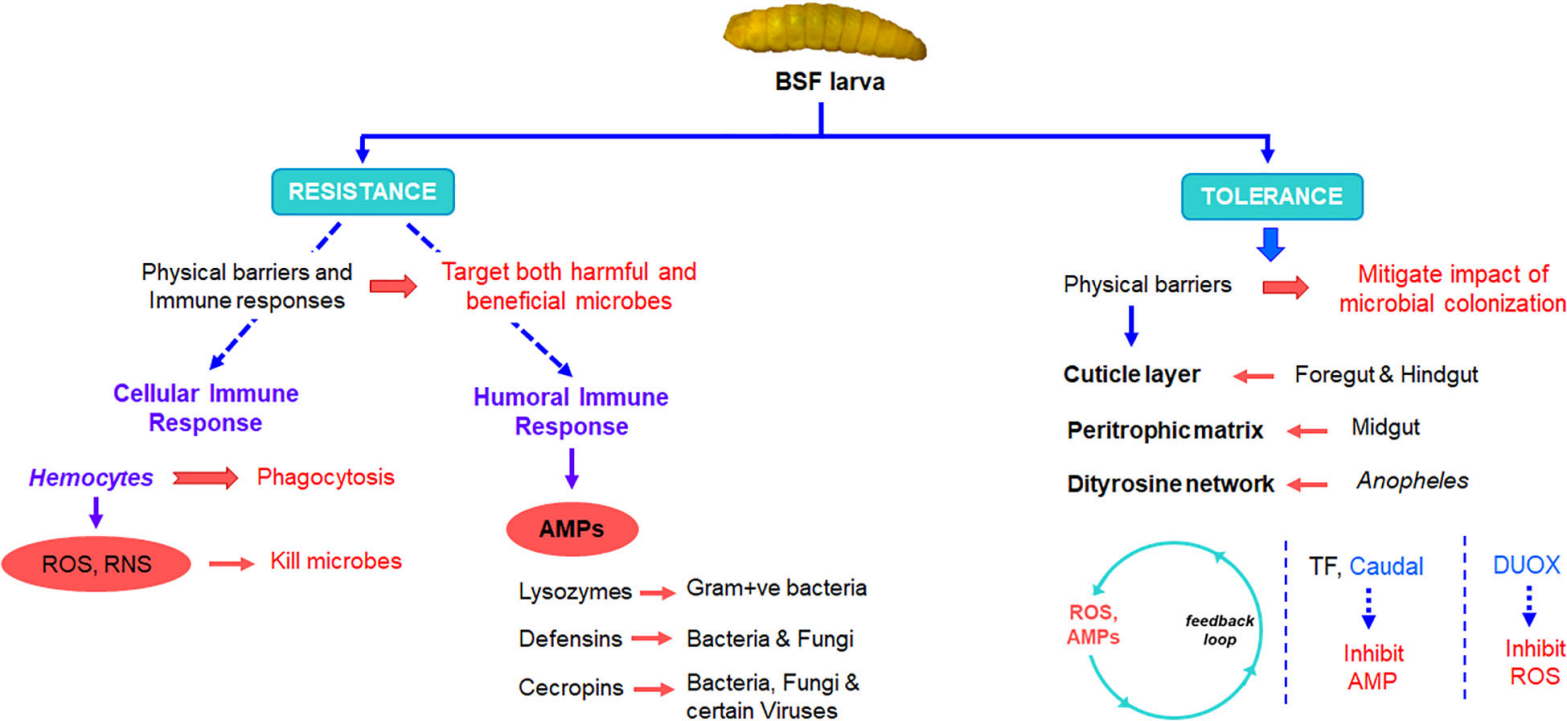
Role of insect immune system in shaping gut microbiota.

The gut of BSF larvae is a complex and highly specialized system that plays a crucial role in their unique feeding behavior and nutrient metabolism. The BSF larval gut consists of several distinct regions, namely, the foregut, the midgut, and the hindgut, each adapted for specific functions in digestion, nutrient absorption, and waste excretion (Eke et al., 2023) (**Figure 2a**). The foregut serves as primary site for food intake and mechanical breakdown of ingested material. Following the foregut, the midgut represents the main site of enzymatic digestion and nutrient absorption. The midgut epithelium is lined with specialized cells called enterocytes, which secrete digestive enzymes such as proteases, lipases, and carbohydrases to break down complex nutrients into simpler forms that can be absorbed. Waste products and undigested material are then passed into the hindgut, where water reabsorption occurs, and feces are compacted before excretion. Naturally, all three-region of host insects harbor different bacterial communities. The midgut of BSF larvae is further differentiated into anterior midgut, middle midgut, and posterior midgut, and are characterized by distinct physicochemical, biological and digestive activities (Bruno et al., 2019; Eke et al., 2023). The anterior midgut is said to be mildly acidic, followed by a strongly acidic middle region, and an alkaline posterior region (Bruno et al., 2019). The pH variation within the midgut of BSF larvae plays a crucial role in shaping the microbial load and diversity within this digestive compartment (**Figure 2b**). This pH gradient creates distinct microenvironments that influence the composition and activity of microbial communities residing within the midgut. The diverse microbial community within the midgut aids in the breakdown of complex organic substrates into simpler compounds, facilitating nutrient absorption by the larvae. However, fluctuations or deviations from the optimal pH range within the midgut can disrupt microbial communities and affect their diversity and composition. This is attested by (Bruno et al., 2019) who observed variances in bacterial load and density in different midgut regions of BSF larvae. The bacterial load and diversity within the larval midgut exhibited contrasting patterns along its length. Specifically, the bacterial load tended to be higher in the posterior midgut compared to the anterior midgut. Conversely, microbial diversity decreased progressively from the anterior to the posterior midgut regions. This phenomenon was attributed to the elimination of most of the bacteria in the anterior and middle region due to combinatorial activity of extreme pH, antimicrobial peptides, lysozymes, and the digestive enzymes (**Figure 3**) (Capo et al., 2019; Lemaitre and Miguel-Aliaga, 2013). These factors collectively create an inhospitable environment for bacteria, leading to a decline in microbial diversity as the contents move through the larval midgut. Once the remaining bacteria reaches the posterior midgut, they proliferate using the available nutrients leading to higher bacterial load. Similar to BSF, midgut of other dipteran insects also display comparable physiochemical distinctions (Barroso et al., 2019; Lemaitre and Miguel-Aliaga, 2013; Overend et al., 2016).

## 4 Functional roles of gut microbiota

**Figure 4:**
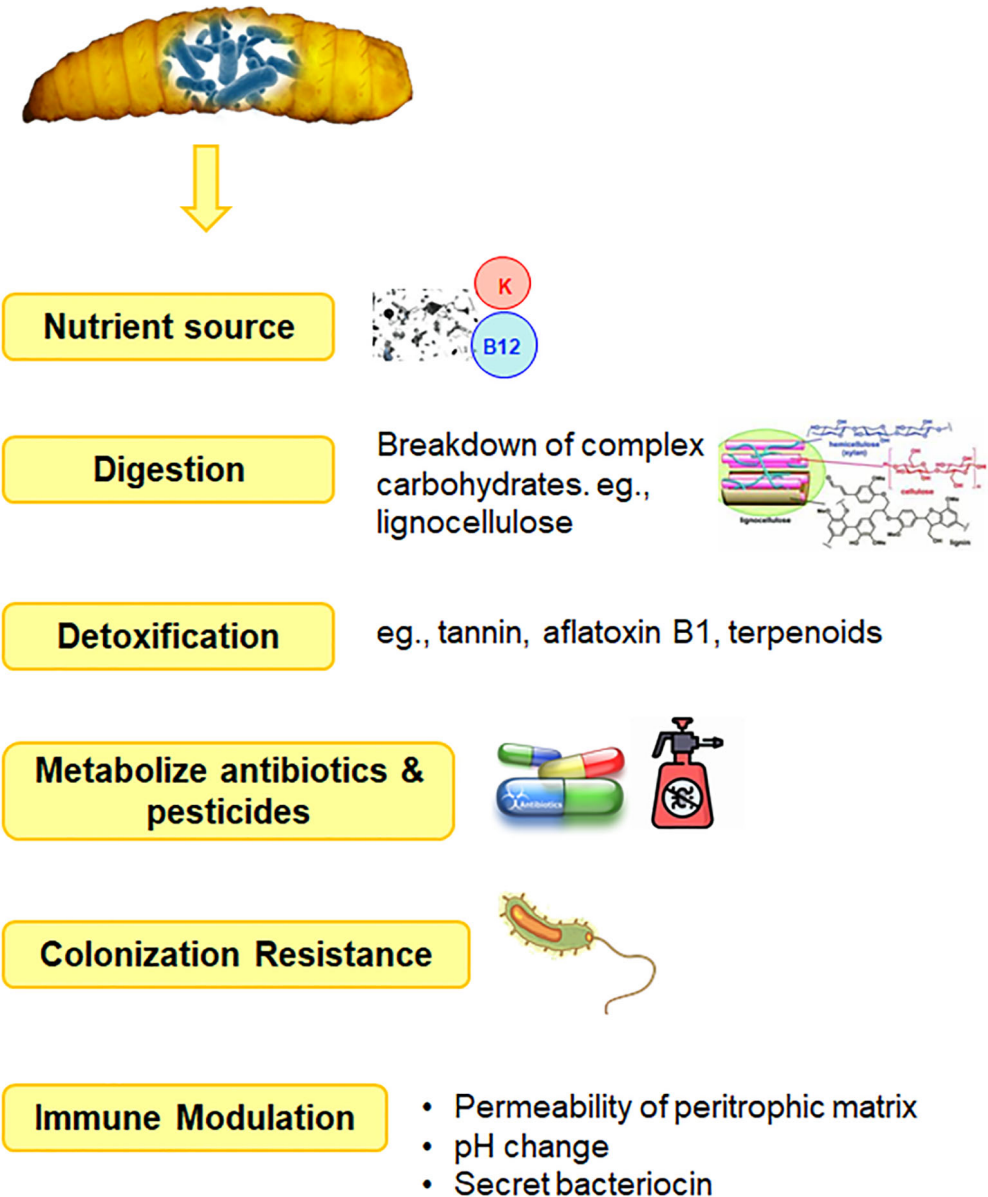
Functional role of gut microbiota.

The gastrointestinal tract of BSF, particularly the larvae, exhibited notable enzymatic activity in terms of amylase, lipase, and protease enzymes (De Smet et al., 2018). The gut extracts from larvae contained significantly higher levels of these digestive enzymes compared to extracts from their salivary glands, with the latter exhibiting less than 10% of the total enzyme activity observed in the gut. The digestive enzymes detected in the gut also showed higher levels of activity compared to enzymes found in the salivary glands. These findings suggested that the larval gut of BSF is a hub for enzymatic activity crucial for the breakdown and digestion of various nutrients present in their diet. Further, the metabolic reactions occurring within the insect gut were correlated with the collective genome of its microbiota, rather than solely by the insect’s own genome. This highlighted the intricate relationship between insects and their gut microbiota, where the microbiota contributes significantly to the host’s physiology and metabolism. The gut microbiota was said to aids in the breakdown and digestion of complex carbohydrates, proteins, and other nutrients present in the insect’s diet, providing essential metabolites and nutrients to the insect host. Jing et al. (2020) observed that the primary function of gut bacteria is to provide essential nutrients, with digestion and detoxification following closely behind in importance (**Figure 4**). In doing so, the gut microbiota might also influence host development, immune function, and overall health.

Bacteria passing through the gut of insects serve as a significant nutrient source, playing a dual role as both food and potential contributors to the insect’s microbiota (Engel and Moran, 2013). These bacteria can readily be digested in the gut and utilized as nutrients by the insect host, a concept often referred to as ‘nutritional bacteria’. Studies have suggested that lysozyme enzymes expressed in the gut of insects, primarily play a role in facilitating the breakdown of bacterial cell walls, thereby releasing nutrients that can be absorbed by the insect for nutrition rather than serving an immune effectors (**Figure 3**) (Karasov and Douglas, 2013). Many other insects have evolved specialized mechanisms, such as harboring endosymbiotic bacteria within specialized cells or organs, where these symbionts play a crucial role in providing essential amino acids, vitamins, and cofactors to their hosts (Cornwallis et al., 2023). These endosymbionts contribute to the overall nutritional ecology of the insect, supplementing their diet with nutrients that may be deficient in their primary food sources. Consuegra et al. (2020) investigated the nutritional contributions of two major gut symbionts of *Drosophila* larvae, *Lactobacillus plantarum* and *Acetobacter pomorum*. They found that these bacteria functionally compensate for the host’s inability to synthesize certain essential compounds by either supplying a metabolic intermediate or derivative of the required nutrient directly to the host. Additionally, they have the capability to absorb, concentrate, and transport trace amounts of micronutrients. Previous research has demonstrated that *L. plantarum*, in particular, promotes intestinal peptidase expression (Erkosar et al., 2015). In nutrient-poor conditions, where the larval diet may lack sufficient protein or other essential nutrients, the presence of gut microbiota that enhance peptidase gene expression becomes particularly advantageous by enhancing the expression of these genes. Thus, gut bacteria can increase the production and activity of peptidases in the larval intestine, which in turn, leads to improved protein digestion and amino acids availability. This has further been elaborated by Grenier (2021) who suggested that rRNAs and/or tRNAs produced by *L. plantarum* may activate GCN2 kinase in the host’s gut, thereby facilitating the host’s physiological adaptation to amino acid imbalance and promoting growth. These findings underscore the multifaceted ways in which gut bacteria can support host nutrition. By providing essential nutrients directly or by modulating host gene expression and enzymatic activity, symbiotic bacteria play a crucial role in ensuring the nutritional well-being and development of their insect hosts.

Wertz and BéChade (2020) delved into mechanism of how gut microbiome contributes to the breakdown of complex carbohydrates. Their dietary constituents of herbivorous insects, such as termites and honeybees, primarily consist of plant-based lignocellulose and other polysaccharides, which are indigestible by the insect’s own enzymes. It has been reported that such non-digestible dietary compounds often reach the insect hindgut, where specialized gut bacteria possess the enzymatic machinery required to break down these complex carbohydrates into simpler sugars and other metabolites (Schmidt and Engel, 2021). This process of microbial degradation and fermentation not only enables gut bacteria to meet their own energy and nutrient needs but also produces metabolites, such as certain vitamins and amino acids, that get absorbed and utilized by the insect host to supplement its nutritional requirements. Lower termites host a diverse community of protists and bacteria—comprising over 400 species per gut—that collaborate with the host’s cellulases to produce acetate, a vital nutrient for the termite (Wertz and BéChade, 2020). This enzymatic activity raises the intriguing possibility that microorganisms residing in the insect gut may sometimes play a role in detoxifying and enhancing the availability of nutrients in the insect’s diet rich in tannins, terpenoids or even mycotoxins (Berasategui et al., 2017; De Smet et al., 2018; de Las Rivas et al., 2019; Govindarajan et al., 2016). The remarkable capacity of BSF larvae to degrade xenobiotics has significant implications for waste management and environmental remediation. The gut microbiota of BSF possess enzymes that can metabolize complex organic molecules, including antibiotics and pesticides, breaking them down into simpler compounds that are more readily absorbed or degraded (De Smet et al., 2018).

The gastrointestinal tract, being continuously exposed to the environment, is susceptible to pathogenic threats that can either colonize the gut, leading to inflammation, or utilize it as a gateway for systemic infections. However, the gut microbiota has a protective role for the host against such pathogenic assaults, in a phenomenon known as colonization resistance (Schmidt and Engel, 2021). This resistance is mediated through various mechanisms that operate within the gut ecosystem (Engel and Moran, 2013). Firstly, commensal or mutualistic microbes can compete with potential pathogens for limited nutrients within the gut environment, thereby inhibiting their growth and establishment. These beneficial microbes occupy niches within the gut, leaving fewer resources available for invading pathogen and thereby impeding their colonization (Dillon et al., 2005; Van Elsas et al., 2012). The presence of commensal or mutualistic microbes can prime the host’s immune system, enhancing its ability to recognize and respond to pathogenic threats effectively. The gut microbiota are able to induce alterations in the physicochemical environment of the insect gut, impeding the colonization of potential pathogens. Another mechanism by which gut microbiota enhance the immune competence of the host is by adaptations for recognizing and responding to invading pathogens. An example of a bacteriocin that directly combat pathogenic invasion is *Enterococcus mundtii*, a prevalent symbiotic bacterium found in the gut of the Egyptian cotton leafworm *Spodoptera littoralis* (Shao et al., 2017). *E. mundtii* actively produces a stable class IIa bacteriocin known as mundticin KS that specifically exhibits inhibitory activity against invading bacteria without affecting other resident gut microbes. The secretion of mundticin KS by *E. mundtii* creates a protective barrier within the gut environment, limiting the expansion of potential pathogens and facilitating the normal development of the host gut microbiota. Moreover, this constitutive production of antimicrobials not only benefits the host by maintaining gut health but also confers a competitive advantage to *E. mundtii*, contributing to its dominance within the insect gut.

## 5 Cross species comparisons

The microbiota of BSF exhibit both shared and distinct features when compared to other waste-degrading insects such as mealworms (*Tenebrio molitor*) and termites. All three insect groups contribute significantly to organic matter degradation and nutrient cycling, largely due to their symbiotic gut microbial communities (Arora et al., 2022; Engel and Moran, 2013; Ndotono et al., 2024). However, the BSF stands out due to its capacity to thrive on a highly variable and microbially dense diet (Bruno et al., 2019; Gold et al., 2018; Siddiqui et al., 2024). This dietary versatility is reflected in dynamic and taxonomically diverse microbiota dominated by *Proteobacteria*, *Firmicutes*, *Actinobacteria*, and *Bacteroidetes*, with microbial composition often shifting in response to substrate type (Bruno et al., 2019; Zheng et al., 2013). Unlike mealworms, which typically consume dry, cereal-based diets and host relatively stable microbiomes optimized for starch and cellulose breakdown (Mamtimin et al., 2023; Yu et al., 2024), BSF larvae exhibit rapid microbiome restructuring that may be involved in the efficient degradation different food sources (Gold et al., 2018). Termites, on the other hand, are highly specialized lignocellulose feeders and maintain a more compartmentalized gut system, densely populated by cellulolytic bacteria and protists that enable the breakdown of woody biomass (Arora et al., 2022; Brune, 2014). Protists were not detected in BSF larvae, possibly due to their highly acidic midgut and rich microbial community, which may contribute to both pathogen tolerance and the ability to convert a wide range of waste substrates (Jeon et al., 2011; Vogel et al., 2018). Altogether, the BSF larvae adaptability, highly efficient biomass conversion rate, and tolerance to environmental fluctuations and pathogens, suggests that the BSF holds a microbiome uniquely suited for large-scale bioconversion systems and a model for studying host-microbe interactions in extreme and fluctuating environments.

## 6 Caveats and challenges in insect microbiome studies

The isolation and identification of insect microbiomes involve both culture-dependent and culture-independent methods. Samples are typically surface-sterilized to remove contaminants (Hammer et al., 2015; Lacey and Kaya, 2007). Then, microbial communities can be cultured on selective media or analyzed directly through DNA sequencing. Culture-independent methods mostly include amplicon sequencing of the 16S rRNA gene (for bacteria) or ITS (for fungi) and shotgun metagenomics for more detailed taxonomic and functional insights. DNA extraction methods must ensure effective lysis of diverse microbial cells or another source (e.g., soil, arthropod body, plant material), and a control to detect contaminations is critical. The bioinformatics tools available for taxonomic classification, diversity, and functional groups analysis are numerous and especially effective for bacterial communities, though, for eukaryotes, different pipelines are recommended (Marcelino et al., 2020; Van Uffelen et al., 2024).

For further validation of the microbiota, especially for the low abundant ones, additional techniques should be considered for gaining complementary insights into microbial localization, abundance, and activity (Lloréns-Rico et al., 2022; Wang et al., 2025). Isolating and identifying the insect microbiome in depth requires employing multiple approaches at once. These approached should include a rigorous sampling protocol driven by the research objectives, sterile techniques, microbial isolations, and a diversity of molecular tools. Culture-dependent methods remain useful for functional assays and bioassays. At the same time, next generation sequencing is more suitable for ecological and taxonomic studies and for the identification of rare microorganisms or non-culturable ones. The technological advances in long-read sequencing, single-cell genomics, and integrative omics (metatranscriptomics, metabolomics) are expected to further enable the studies of complex biological systems such as insect-associated microbiomes. Recently, Next Generation Sequencing studied the virome of BSF, highlighting the power of those molecular tools for non-culturable organisms such as viruses (Pienaar et al., 2025).

## 7 Future perspectives

The intricate interaction between BSF and its associated microbiota raises important research questions related to its ecology, behavior, dietary plasticity, and tolerance to pathogens and xenobiotics. One promising yet underexplored avenue to address these questions is microbiota engineering. Targeted manipulation of the BSF microbiome represents a novel strategy to enhance key biological traits, including immunity and reproductive capacity, as well as to improve bioconversion efficiency—an essential parameter for the optimization of large-scale organic waste processing systems. However, empirical data supporting this approach remain limited.

Genetic and ecological engineering strategies may facilitate the enrichment or stabilization of core microbial taxa responsible for specialized metabolic functions, such as nitrogen fixation, cellulose degradation, or lipid metabolism, tailored to specific input substrates. Effective implementation of these strategies requires a comprehensive understanding of host–microbiome interactions, microbial community stability, and the functional redundancy within the ecosystem to prevent unintended disruptions.

Controlled modulation of the microbiota stands at the forefront of BSF biotechnology, offering potential applications that include improved substrate degradation, enhanced nutritional quality of larval biomass, and the generation of higher-value by-products such as frass and insect-derived proteins for use in agriculture. The strategic introduction of functional microbial consortia—such as proteolytic and cellulolytic bacteria—into larval rearing systems holds significant promise for boosting biomass yield and substrate conversion rates.
